# The role of fermented foods in maternal health during pregnancy and infant health during the first 1,000 days of life

**DOI:** 10.3389/fnut.2025.1581723

**Published:** 2025-06-05

**Authors:** Arun Pandiyan, Manoj Gurung, Bharath Kumar Mulakala, Sathish Kumar Ponniah, Laxmi Yeruva

**Affiliations:** ^1^Microbiome and Metabolism Research Unit, USDA-ARS, SEA, Little Rock, AR, United States; ^2^Arkansas Children’s Nutrition Center, Little Rock, AR, United States; ^3^Department of Agriculture, University of Arkansas at Pine Bluff, Pine Bluff, AR, United States; ^4^Texas A&M AgriLife Institute for Advancing Health Through Agriculture, College Station, TX, United States

**Keywords:** fermented foods, pregnancy, infant, brain, immune system, gut microbiota

## Abstract

Fermented foods are a good source of nutrition, with microbiota and metabolites that can positively influence consumer health. With the increasingly negative health outcomes from using low-quality diets like processed diets, functional products like fermented foods are getting more attention than ever. All cultures of the world consume some kind of fermented foods. Extensive literature outlines positive health and clinical outcomes associated with fermented foods, yet most data are associative and lack longitudinal studies. This review explores the role of fermented foods during pregnancy and its subsequent impact on maternal and infant health, especially in the first 1,000 days of life. In this review, we have summarized the literature on fermented foods from preclinical and clinical studies that evaluated the impact of maternal consumption of fermented foods on mothers and offspring microbiota, immune system, and brain health outcomes. We also discussed existing knowledge gaps on maternal-child dyads and mechanistic studies needed to provide better scientific evidence to promote fermented foods consumption.

## 1 Overview

A broad range (~5,000 varieties) of traditionally produced fermented foods are consumed worldwide (1, 2). Though in recent years there has been a resurgence in the consumption of fermented foods due to proposed health benefits, advantages as a low-tech, resource-efficient processing, and concerns about food waste and environmental impact (3, 4), expansion in processing technologies, industrialization, and commercialization of food production has reduced the consumption of fermented foods overall, especially in Western countries (5). Many types of fermented foods are made and consumed, including dairy, plant, cereal, vegetable-based and alcoholic beverages (2, 6). Though alcoholic beverages fall under the larger umbrella of fermented foods, they are detrimental to maternal health. Given that the objective of our review is to explore the potential beneficial effects of maternal consumption of fermented foods, we exclude alcoholic beverages from our review.

During pregnancy, nutritional needs increase to support the growth and development of the fetus and to improve the tissue reserves and metabolic demands of the mother. These increased needs include energy, macronutrients, and specific micronutrients (7). Fermented foods can offer greater nutritional benefits than their unfermented counterparts as the microorganisms involved can break down complex compounds to produce multiple byproducts, such as vitamins and other micronutrients. For example, fermented grains are nutritionally superior to unfermented grains, as fermentation releases nutrients trapped within plant structures and cells (8). In addition, even after cooking, some nutrients trapped in food may remain inaccessible to the human digestive system (9). This can be countered by fermentation, which can break down the indigestible coatings and cell walls chemically and physically, thereby releasing essential nutrients. Cellulose, hemicellulose, and related polymers are indigestible by humans. During fermentation, enzymes from microorganisms can split complex carbohydrate molecules, breaking them down into more digestible simple sugars and sugar derivatives (10). Moreover, fermented dairy products serve as a source of probiotics, prebiotics, and bioactive compounds and are actively promoted as functional foods for their nutritional and therapeutic values (11). In addition to increasing nutrient accessibility, fermentation can also reduce the presence of antinutrients such as phytic acid, trypsin inhibitors and tannins, enhancing the bioavailability of essential minerals such as iron, protein, and simple sugars (12). Phytic acids can reduce the bioavailability of minerals and significantly impact pregnant women, lactating mothers, and infants, especially when large amounts of cereal-based foods are consumed (11, 13).

David Barker’s assertion that *“Much of human development is completed during the first 1,000 days after conception”* laid the foundation for a significant shift in our understanding of early life influences on long-term health (14). This concept evolved into the more comprehensive Developmental Origins of Health and Disease (DOHaD) theory (15, 16). “The first 1,000 days” are considered to impact a child’s health and future disease risk. In recent years, there has been a growing focus among policymakers on the critical importance of early childhood development. Specifically, two key periods have garnered significant attention: the “first 1,000 days” and the “0–3 years” age range. These timeframes are increasingly recognized as crucial windows of opportunity for positively shaping a child’s future health outcomes. Maternal nutrition, dietary behavior’s, and environment also play a vital role in shaping the first 1,000 days of life, resulting in “nutritional programming” (17). While micronutrient, mineral, and probiotic supplementation have recently gained increased attention in maternal nutrition, there is immense potential in focusing on traditional nutrient-dense foods to meet these dietary needs during pregnancy without increasing economic and accessibility burdens on the families. The maternal microbiome plays a critical role in modulating the infant gut microbiome, immune system and its subsequent role in the gut-brain axis, and fermented foods inherently act as probiotics. Given the critical role of nutrition during pregnancy, the influence of the maternal microbiome on infant development, and the promising potential of fermented foods to improve health outcomes, this review aims to highlight the existing literature on the role of fermented foods in modulating gut microbiome, immune function, and brain health. Further, this review evaluates the existing evidence and addresses gaps in the gut-immune-brain triad in mother and offspring with respect to maternal fermented foods intake. We also propose that the utilization of fermented foods during pregnancy may increase beneficial health outcomes in both mothers and infants, especially during the first 1,000 days of life.

## 2 Methodology

### 2.1 Objectives and review design

We evaluate the current knowledge of fermented food’s impact on maternal and infant health. We relate maternal fermented food intake with alteration in the mother’s and offspring’s gut microbiome and subsequent effects on the infant’s immune functions and brain health. For this review, we searched literature databases using the following terms: “fermented foods/gut microbiota,” “infant microbiome/fermented foods,” “fermented foods/maternal outcomes,” “pregnancy complications/fermented foods,” “infant brain/fermented food,” “fermented foods/immune functions” in “humans,” “mice,” “porcine,” and “primate” in different combinations, focusing on studies published from the year 2000 until December 2024. This comprehensive literature search was conducted across multiple academic databases, including PubMed, Google Scholar, Web of Science, Scopus, Cochrane, and Science Direct. Both observational and experimental study designs were included in this review. Since the first 1,000 days are considered the “Golden Opportunity” (18, 19) to stimulate a child’s development and growth, we limit our inclusions to infant health during the first 1,000 days (<3 years) alone. We also limit our literature search to whole fermented foods, not the individual components within fermented foods, such as metabolites or nutrients. Further, we discussed the existing knowledge gap on the role of fermented foods in maternal-child health. This review addresses the research question, *“Can incorporating fermented foods in the maternal diet produce positive health outcomes in mothers and infants?.”* With this review, our objective is not to narrate the existing literature in the adult population but to contribute to a better understanding of the areas within maternal fermented food intake and infant gut, immune and brain outcomes where there is a knowledge gap.

#### 2.1.1 Inclusion criteria

Studies published in peer-reviewed journals, cohort studies in humans reporting consumption of traditional, homemade, or commercially available fermented foods during pregnancy using food-frequency questionnaires including 24-h dietary recall and self-reported formats; intervention studies in humans where pregnant women were fed specific fermented foods; clinical studies examining the effects of maternal fermented food consumption on offspring’s health; animal studies involving intervention with specific fermented foods during pregnancy, and studies exploring the outcomes based on the transition from normal diet to maternal fermented food diet.

#### 2.1.2 Exclusion criteria

Studies focusing on fermented foods in addition with any probiotic, micronutrients or mineral supplementation, with exercise, and vaccine as these factors could confound the specific nutritional, immunological and microbial effects of fermented foods, influence biomarkers such as serum insulin affecting gestational diabetes independently of diet, or interfere with the immunomodulatory effects; intervention studies in humans on the effect of fermented foods without any direct relationship to pregnancy or maternal diet; animal studies examining fermented foods outside the context of maternal nutrition or offspring health; longitudinal studies focusing on maternal consumption of fermented foods and health outcomes in children extending beyond 3 years of the child’s age; review articles, opinion pieces, or non-empirical publications; studies with significant methodological flaws or insufficient data reporting.

## 3 Role of fermented foods in ameliorating pregnancy-related complications

Nutritional inadequacy in the form of malnutrition can lead to pregnancy-related complications such as infertility, gestational diabetes, maternal hypertension, preterm birth, and asthma (20), so it is not surprising that most maternal-related deaths occur in regions and groups facing increased food insecurity (21, 22). With inconclusive and contradictory evidence from supplementation studies during pregnancy (23, 24), the ideal scenario would be to strengthen nutritional intake through foods. Fermented foods, rich in bioavailable nutrients and probiotics, could offer a practical dietary strategy to address these deficiencies, especially in low-income and middle-income countries. Fermented foods such as miso soup, yogurt, cheese and fermented soybeans have been shown to reduce the risk of pre-term birth (25). In this regard, it is essential to explore the role of fermented foods in ameliorating pregnancy-related complications. Enterobacterial microbiota differences found between groups of pregnant women consuming fermented foods and control groups, respectively, were hypothesized to reduce pre-term birth cases, by reducing pro-inflammatory Enterobacteriaceae associated with infection or immune dysregulation (25). In another study, Mexican women who reported consuming yogurt during the last 3 months of pregnancy had a decreased risk for pre-term birth, suggesting that prenatal yogurt consumption may reduce the risk of pre-term birth among non-overweight pregnant women. The authors suggest this effect could be due to probiotic strains in yogurt with anti-inflammatory properties (26). However, this was a questionnaire-based prospective study and entirely observational thus, observations should be interpreted cautiously.

Though the global prevalence of anxiety and depression during pregnancy varies between studies, especially across developing and developed countries (27), it is now widely accepted that maternal anxiety, stress and depression can have a lasting effect on both the mother and the baby (28). In addition, the impact of prenatal stress is further exacerbated by nutrient restriction (29). A high-quality maternal diet, as defined by the presence of fruits, vegetables, fishes and whole grains, has been shown to lower the risk for prenatal depressive symptoms as compared to the consumption of other food groups such as refined grains, fast foods, and energy drinks (30, 31). Only a few studies have explored the consumption of fermented foods in maternal depressive symptoms and stress. In a Japanese cohort, consumption of seaweed, yogurt, tofu, fermented soybeans and miso soup was found to be negatively associated with depression during pregnancy, which could mitigate the impact of maternal stress on the offspring (32). On the contrary, a Japanese cohort of around 9,030 pregnant women showed no association between maternal fermented food diet and psychological distress (33). Also, the mechanisms behind these effects have not been identified.

Gestational diabetes (GDM) is another widely prevalent pregnancy complication. According to the Centers for Disease Control and Prevention (CDC), the percentage of mothers with gestational diabetes in the United States increased from 6% in 2016 to 8.3% in 2021 (34). Recent clinical studies have shown substituting higher complex carbohydrates for simple carbohydrates can help control maternal glycemia and reduce postprandial glucose. In a randomized crossover study, two distinct diets, one rich in complex carbohydrates with less fat, and the other lower in carbohydrates with more fat were evaluated by continuous glucose monitoring (CGM) over 72 h in 16 women with gestational diabetes mellitus (GDM). The results indicated that the diet higher in complex carbohydrates and lower in fat maintained blood glucose levels below established treatment goals while also reducing postprandial free fatty acid concentrations (35). Fermented foods derived from grains, legumes, or starchy vegetables can be sources of complex carbohydrates. Further, the ingestion of fermented foods, especially plant-based, can increase the digestibility of complex carbohydrates by degrading starch into more digestible oligosaccharides (36). For example, sourdough-leavened breads are more digestible than conventionally made breads due to the pre-breakdown of gluten by lactic acid bacteria (LAB) through proteolysis during fermentation (37). Among pregnant women, soy-bean oligosaccharides were found to alleviate insulin resistance among those with gestational diabetes (38), and several studies have suggested fermented food supplementation can reduce the risk of diabetes through antioxidant and anti-inflammatory properties (39). In a study involving 70 pregnant women of singleton pregnancy, subjects who consumed probiotic yogurt had higher levels of erythrocyte glutathione reductase (GR), an enzyme linked with insulin sensitivity, than those who consumed conventional yogurt (40). Another study reported that daily consumption of probiotic yogurt by pregnant women for 9 weeks stabilized serum insulin levels, suggesting potential prevention of pregnancy-induced insulin resistance (41). A study comparing patients with gestational diabetes mellitus and healthy pregnant women found that consumption of white wheat bread resulted in 45.5% higher insulin secretion and a 9.6% increase in first-hour postprandial blood glucose levels compared to sourdough whole grain bread in both groups (42). Among women with gestational diabetes, probiotic yogurt containing the probiotic strains *Lactobacillus acidophillus* and *Bifidobacterium animalis* (formerly known as *Bifidobacterium lactis*), reduced the risk of gestational diabetes (41, 43–45).

Gestational hypertension is considered a risk factor for preeclampsia, preterm birth, and low birth weight. Dietary strategies, including consumption of fermented foods to prevent hypertension, have yielded positive results. Fermentation of milk by lactic acid bacteria can produce various bioactive peptides with hypotensive properties (46). These peptides maintain their activity throughout the digestive process and can be absorbed into different organs and tissues via the bloodstream. One group of these peptides is angiotensin-converting enzyme inhibitory peptides (ACE-I), which has garnered significant interest for their potential use in managing hypertension. Both clinical and animal studies have demonstrated the blood pressure-lowering effects of certain fermented milk products containing these peptides (47). Among yeasts, two strains, namely *Pichia kudriavzevii* KL84A and *Kluyveromyces marxianus* KL26A from Kumis, a traditional fermented Colombian milk, showed the presence of these ACEI peptides (48). In one study, lactic acid bacteria with high ACEI activity, *Lactiplantibacillus plantarum B* (formerly known as *Lactobacillus plantarum*), *Lactobacillus gasseri A*, *Lactiplantibacillus plantarum* R5 (LPR5), and *Lactiplantibacillus plantarum* R7 (LPR7), showed antihypertensive effect in rat models with pregnancy-induced hypertension (49). In general, though large cohort studies and countable intervention studies have found a link between maternal fermented foods consumption and reduced risk of pregnancy complications, more in-depth clinical studies with fermented foods intervention among pregnant women would inform preventive strategies for reducing pregnancy-related complications.

## 4 Can maternal consumption of fermented foods modulate the gut microbiome of mothers and offspring?

Human gut microbiota has gained much attention due to increasing evidence of compositional and structural changes of gut microbiota playing a vital role in various aspects of human health, including immune, metabolic, and neuro-behavioral traits (50). Multiple factors, such as environment, genetics, infection, and mode of delivery, can influence gut microbiome composition. However, diet is also one of the most important variables in modulating gut microbiota composition throughout life (51).

Fermented foods can modulate the gut microbiota across the human lifespan due to the microbial strains present in the fermented foods (Table 1). Published literature suggests that one of the reasons for the beneficial health effects of fermented foods could be attributed to the promotion of probiotic strains and reduction in “pathobionts”—microorganisms that are non-pathogenic under ideal gut conditions but can turn pathogenic under adverse conditions (52). In a study, consumption of fermented milk (250 g/d) for 4 weeks improved irritable bowel syndrome status in women by increasing the short-chain fatty acids (SCFA) production and decreasing the abundance of the pathobiont *Bilophila wadsworthia* (53). Animal studies have shown that consumption of fermented milk products, such as Kefir, increased the abundance of beneficial gut bacteria, such as *Lactobacillus, Lactococcus, and Bifidobacterium* (54, 55). Furthermore, kefir may help reduce intestinal permeability and improve tight junction function of the gut in adults and older populations (56). Similarly, a dose-dependent response of *S. thermophilus* and *B. animalis* abundances was observed with yogurt consumption in the older human population. It was observed that high yogurt consumption (more than 5 times/week) showed higher levels of *Bifidobacterium animalis* and *Streptococcus thermophilus* than low yogurt consumption (1–5 times/week) (57). Similarly, the abundance of *Bacteroides, Dorea, Prevotella, and Faecalibacterium prausnitzii* was higher in the fecal samples from healthy adults consuming grain and vegetable-based fermented food compared to the non-consuming group (58). Among these microbes, *Bacteroides, Dorea* and *Faecalibacterium* are known to have positive health effects such as immune modulation, short-chain fatty acids production, better response to immunotherapy, and improved glucose homeostasis (59–62). In a cohort of Korean women, the abundance of 34 microbial species in the gut significantly differed between groups with low (15 g/day) and high (150 g/day) Kimchi consumption. The abundance of beneficial bacteria, such as *L. acidophilus*, *Levilactobacillus brevis* (formerly known as *Lactobacillus brevis*), *Bifidobacterium breve*, *Lactobacillus amylolyticus*, *Companilactobacillus mindensis* (formerly known as *Lactobacillus mindensis*), *Limosilactobacillus reuteri* (formerly known as *Lactobacillus reuteri*), and *L. mesenteroides* was significantly higher in the high Kimchi group (63).

**Table 1:** Commonly consumed fermented foods, their origin and microbial strains present in ethnic foods.

Fermented food	Microbiome present	Region of origin	References
*Budu* (anchovy sauce)	*Micrococcus luteus, L. delbrueckii, Pediococcus pentosaceus, P. acidilactic and Staphylococcus arlettae*	*Malaysia*	(98)
*Cultured buttermilk*	*Lactococcus, Lactobacillus, Streptococcus,* and *Leuconostocs*	India	(99)
*Greek Yogurt*	*L. delbrueckii* subsp. *bulgaricus* and *S. thermophilus*	Middle East	(100)
*Jiang Gua* (pickled cucumbers in soy sauce)	*Lactobacillus paraplantarum, L. pentosus, L. plantarum, Leuconostoc mesenteroides, Leuconostoc lactis, L. lactis subsp. lactis, Weissella hellenica and W. cibaria*	Taiwan	(101)
*Kimchi* (fermented vegetables)	*Lactobacillus acidophilus, Leuconostoc mesenteroides, L. plantarum* and *L. sakei*	Korea	(102)
*Kefir*	*Lactobacillus kefiranofaciens* subsp. *Kefirgranum, Streptococcus thermophilus, L. delbrueckii* subsp. *bulgaricus, L. kefiranofaciens* subsp. *kefiranofaciens, L. helveticus, L. acidophilus*; *Lactococcus lactis* subsp. *lactis,* and *L. lactis* subsp. *cremoris*	North Caucasus	(103)
*Sauerkraut* (fermented cabbage)	*Lactobacillus plantarum, Leuconostoc. mesenteroides* subsp. *Mesenteroides, L. brevis*	China	(104)
*Suan Tsai* (Chinese sauerkraut)	*Tetragenococcus halophilus and Pediococcus pentosaceus*	Taiwan	(105)
*Sian Sianzih* (fermented clams)	*Lactococcus lactis, Lactobacillus sakei, and Weissella Hellenica*	Taiwan	(106)
*Tempeh* (fermented soybeans)	*Rhizopus oligosporus, Lacticaseibacillus casei* (formerly known as *Lactobacillus casei*) and *Streptoccocs fuecium*	Indonesia	(107)
*Miso and Koji* (fermented rice and soybeans)	*Aspergillus oryzae, Tetragenococcus halophilus, Zygosaccharomyces rouxii, Bacteroides dorei, L. delbruekii, L. pentosus, Candida prapsilosis and Staphylococcus succinus*	Japan	(108)
*Sourdough*	*L. plantarum, L. fermentum, L. paralimentarius,* and *Lactococcus lactis*	Egypt	(109)
*Natto* (fermented soybeans)	*Bacillus subtilis* var. *natto*		(110)
*Gochujang* (fermented red chili)	*Bacillus velezensis, Zygosaccharomyces rouxii, Candida lactis, Z. rouxii and Z. bailii*	Korea	(111)
*Meju* (fermented soybeans)	*Bacillus sonorensis, Enterococcus darans,* and *E. darans*	Korea	(112)
*Cheese*	*Bifidobacterium infantis, B. longum, B. animalis* ssp. *lactis, B. bifidum, Lactobacillus acidophilus, L. paracasei, L. plantarum, L. casei, L. rhamnosus, Lactobacillus gasseri*	*Inconclusive evidence on origin*	(113)
*Idli* (rice cake)	*Streptococcus faecalis, Leuconostoc mesenteroides, Lactobacillus coryneformis, L. fermentum, L. lactis, L. delbrueckii,* and *Pediococcus cerevisi*	India	(114)
*Koozh* (millet porridge)	*Weisella paramesenteroides* and *L. fermentum*	India	(115)
*Fermented rice*	*Lactobacillus helveticus, L. delbrueckii* ssp. *Delbrueckii, L. pentosus* and *L. curvatus* ssp. *curvatus,*	Asia	(116)
*Kombucha* (fermented black tea)	*Komagataeibacter kombuchae, Gluconacetobacter sacchari, Acetobacter liquefaciens, Z. lentus* and *Z. bisporus*	China	(117)
*Airag* (fermented horse milk)	*L. helveticus, L. kefiranofaciens, Bifidobacterium mongoliense, Kluyveromyces marxianus*	Mongolia	(118)
*Yan Jiang* (fermented ginger)	*Weissella cibaria, L. mesenteroides, Lactobacillus plantarum, L. sakei, Leuconostoc citreum, Leuconostoc garvieae, Pediococcus stilesii, Enterococcus sulfureus* and *Lactococcus lactis* subsp. *lactis*	Taiwan	(119)
*Meekiri* (fermented buffalo milk)	*Limosilactobacillus fermentum, L. curvatus, L. acidophilus, Lactobacillus planatarum, L. helviticus, L. delbrueckii* subsp. *lactis, L. delbrueckii* subsp. *bulgaricus and L. casei* subsp. *casei, S. thermophiles and S, lactis, Micrococcus* spp., *Saccharomyces cerevisiae and Bacillus* spp.	Sri Lanka	(120)
*Poko* (rice)	*Candida versatilis, S. cerevisiae, Pediococcus* spp., *Lactobacillus* spp., *and Rhizopus* spp.	Nepal	(121)
*Kinema* (fermented soybeans)	*Bacillus subtilis, B. licheniformis, B. thermoamylovorans, B. sonorensis, B. cereus, Ignatzschineria larvae, Brevibacillus borstelensis, Corynebacterium casei, Proteus vulgaris, Thermoactinomyces vulgaris, L. fermentum and Ignatzschineria indica, Wallemia canadensis, Rhizopus arrhizus Penicillium* spp., *Mucor circinelloides, Aspergillus penicillioides, A.* spp., *Exobasidium* spp., *Arthrocladium* spp., *and Mortierella* spp.	Nepal	(122)
*Cheonggukjang* (fermented soybeans)	*B. subtilis, B. licheniformis and B. amyloliquefaciens*	Korea	(123)
*Oshikundu* (fermented millet)	*L. delbrueckii* ssp. *delbrueckii, L. curvatus* ssp. *Curvatus, L. plantarum, L. pentosus, L. fermentum, L. lactis* ssp. *lactis*	Namibia	(124)
*Cauim* (fermented cassava)	*Corynebacterium. vitarumen, C. amylocolatum, C. xerosis, Paenibacillus macerans. Bacillus cereus, B. circulans B. licheniformis, B. pumilus, and Lactobacillus pentosus,* and *L. plantarum*	Brazil	(125)

**Figure 1:**
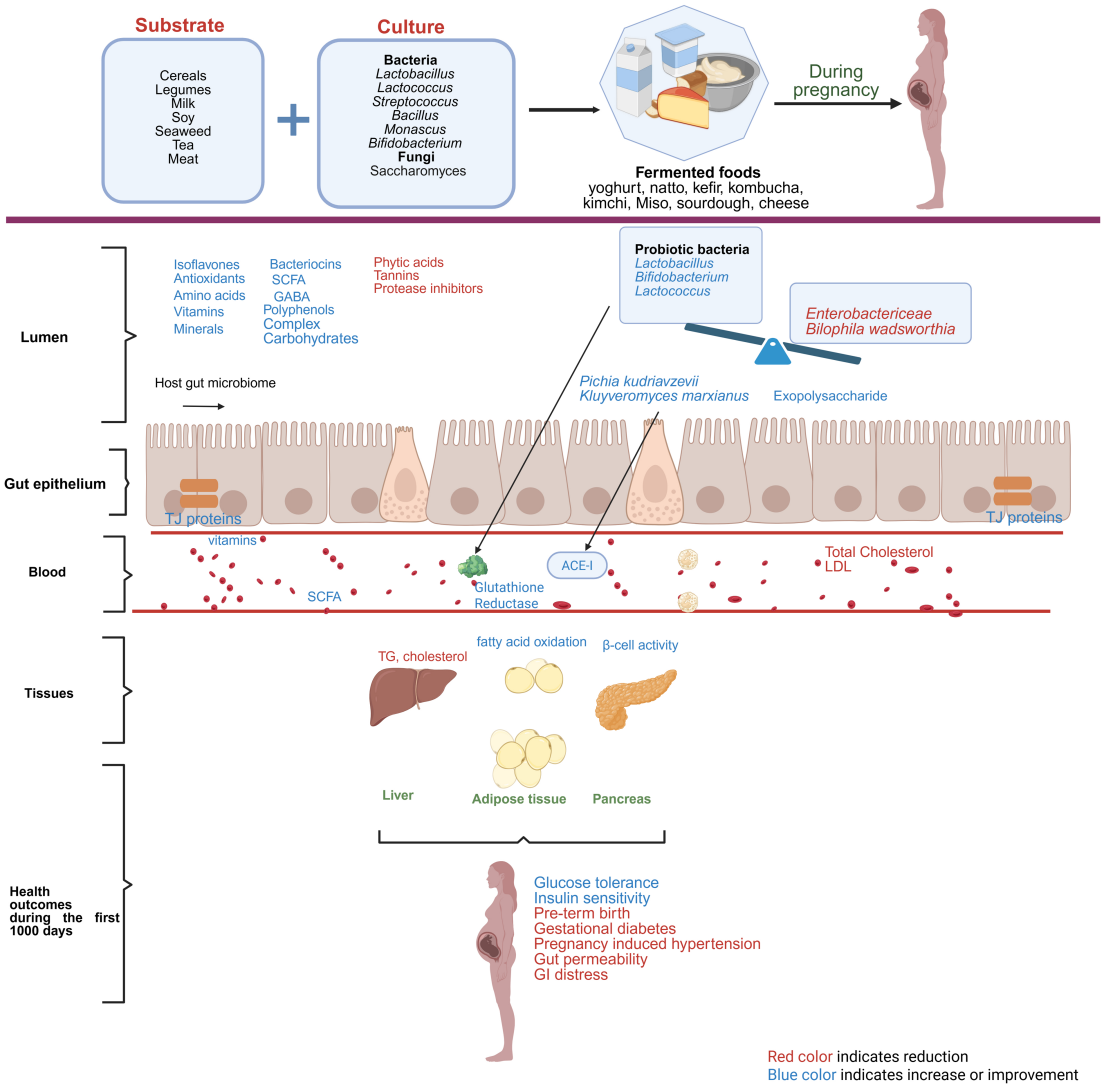
Role of fermented foods in ameliorating pregnancy-related complications. Red color text indicates reduction and blue color text indicates improvement or amelioration due to maternal consumption of fermented foods.

Though, as outlined above, it is known that grain and vegetable fermented foods can modulate gut microbiome across age groups, very few studies have reported on alterations of gut microbial composition during pregnancy and infancy. One study reported an increase in the fecal levels of the beneficial microbe *Bifidobacterium* and a decrease in pathogenic *Enterobacteriaceae* in infants born to mothers who consumed 250 g of yogurt (6 days/week) during pregnancy (between 12 and 24 weeks of gestation), and up to 1 month postpartum (64). Similarly, a study conducted in Wistar rats showed a higher abundance of Bacteroides in the offspring’s (21 days old) gut, following maternal Kefir supplementation (65). The effects of fermented mulberry (FM) supplementation in pigs revealed microbial shifts in both sows and their offspring (66). FM-fed sows exhibited an increased relative abundance of *Bacteroides* compared to the control group, while piglets (offspring) from FM-supplemented sows demonstrated a higher relative abundance of Firmicutes than piglets from the control group, suggesting that maternal dietary intervention with fermented food can influence the gut microbiome composition, not only in the mothers themselves but also in their progeny (66). This finding was supported by another study carried out in mice where supplementation with milk fermented with *Lacticaseibacillus casei* DN-114001 (formerly known as *Lactobacillus casei*) showed higher levels of *Bifidobacteria* in the intestine of mothers during the suckling period and in the newborns after weaning, suggesting a potential transgenerational modulation of the gut microbiota due to fermented food (67). Figure 1 summarizes the role of fermented foods in modulating gut microbiome and ameliorating pregnancy related complications.

## 5 Do fermented foods play a role in modulating maternal and infant immune systems?

The perinatal nutrition status of both mothers and offspring impacts the immunological response and functional maturation of the immune system, and proper dietary patterns and interventions benefit both maternal and offspring’s immune homeostasis. Studies on fermented foods and their effect on modulating the maternal and infant immune system are limited but encouraging. In an intervention study, consuming fermented milk with *L. casei* as a starter culture during the post-partum period decreased milk TNF-*α* levels and reduced the frequency of gastrointestinal symptoms in infants at 2–6 months of age (68). Similarly, in a porcine model, the sows’ consumption of a fermented diet ameliorated the offspring’s colonic inflammation (69). In a study conducted in mice, maternal yogurt intake significantly increased offspring Innate lymphoid Cells 3 (ILC3). It was found that yogurt-derived indole compounds can activate the aryl hydrocarbon receptor (AhR) signaling pathways, promote ILC’s differentiation and proliferation which in turn produces cytokines essential for gut barrier function and protection against infection (70). These findings suggest maternal consumption of fermented foods could benefit offspring’s intestinal immune function. In another preclinical study, when pregnant Swiss albino mice were fed fermented milk containing *Lacticaseibacillus rhamnosus* 5,897 (formerly known as *Lactobacillus rhamnosus*), serum IgG, which neutralizes pathogens and provides passive immunity against infections during early years was significantly elevated in offsprings during the suckling and post-weaning periods (71). Similarly, maternal supplementation of fermented milk with *L. casei* DN-114001 as starter culture, showed significant downregulation in immune cell markers related to macrophages and dendric cells in offsprings, suggesting passive immunity provided by the mother (67). Interestingly, dietary restriction of fermented foods has shown negative effects on the innate immune response, which was reversed partially by the consumption of yogurt (72). These reports suggest the potential of maternal fermented food consumption to modulate offspring’s innate and adaptive immune response.

**Figure 2:**
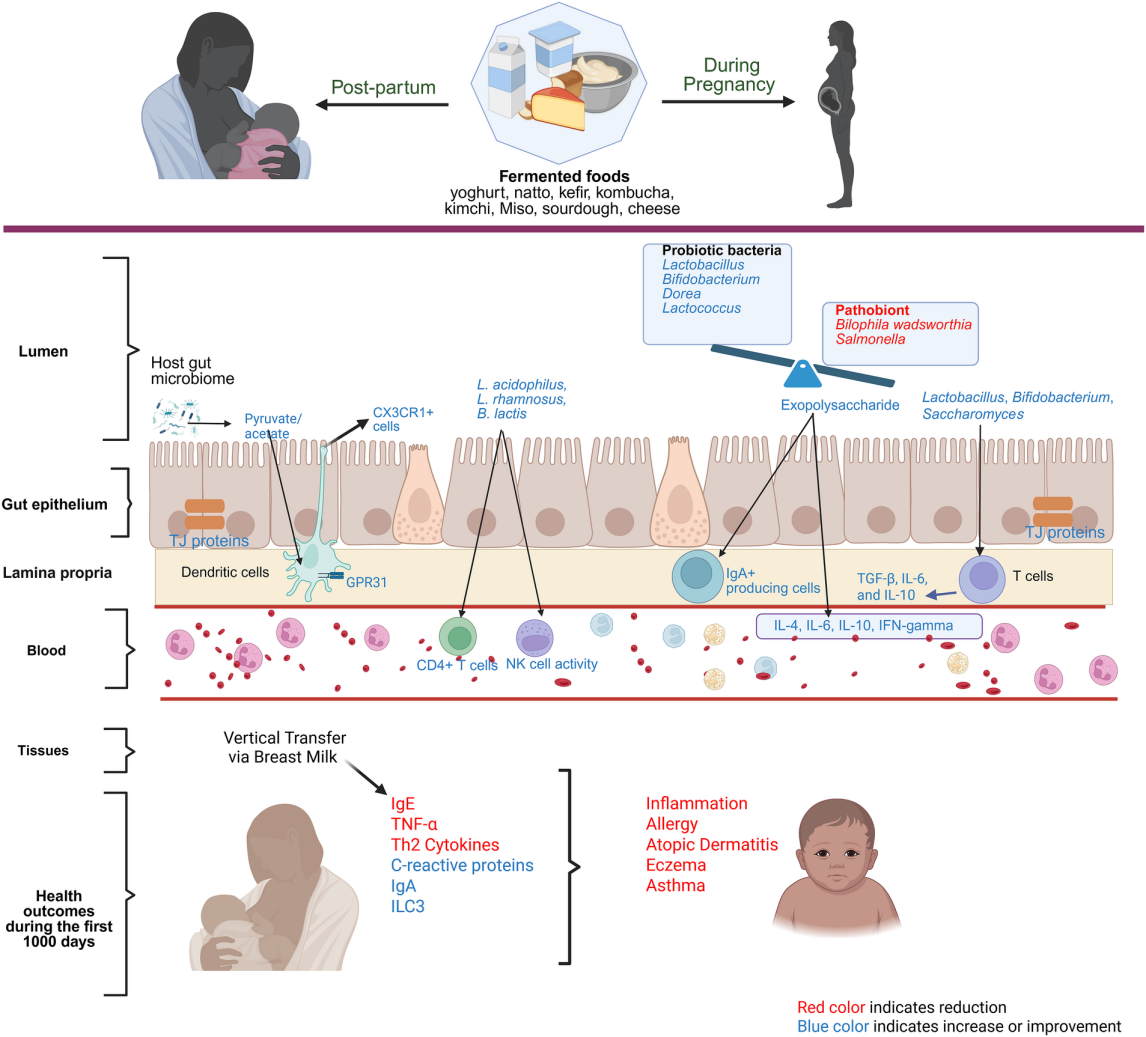
Role of fermented foods in modulating the immune system during pregnancy. Red color text indicates reduction and blue color text indicates improvement or amelioration due to maternal consumption of fermented foods.

The global prevalence of allergic diseases in newborns is on the rise, often linked to early-life immune system development (73). Several human and preclinical studies emphasize the importance of gut microbiome during the first 1,000 days of life, both maternal and infant, in shaping immune development in childhood (74). To exemplify, maternal microbiome influences offspring’s immune tolerance, reducing allergic disease and asthma risks at birth (75). Food Protein-Induced Allergic Proctocolitis (FPIAP) has been associated with lower consumption of fermented foods, such as yogurt, cheese, and tarhana, during pregnancy, highlighting the potential protective role of these foods (76). In a case–control study conducted in Turkey, it was observed that mothers of healthy children had a significantly higher frequency of daily yogurt consumption during pregnancy compared to mothers of children diagnosed with atopic dermatitis between the ages of 2 and 24 months (77). Similarly, a large cohort study from Norway reported an association between the reduction in the risk of atopic eczema in infants at 6 months of age with the maternal consumption of probiotic milk and yogurt during pregnancy, although no clear dose–response relationship was identified (78). Also, in a study conducted in China, both the frequency and quantity of maternal yogurt intake were associated with a dose-dependent reduction in the risk of eczema in infants aged 3 to 6 months. This study reported nearly a 50% decrease in the risk of eczema in infants whose mothers consume yogurt more than three times per week and more than 50 g per day (79). The underlying mechanism is that probiotics promote microbial stimulation, modulating the immune system that supports a balance between T helper 1 (Th1) and Th2 cells, which reduces Th2 cytokines, IgE concentrations, and increases C-reactive protein and IgA levels to prevent inflammation and allergy-related processes (80). Additionally, the anti-inflammatory properties of yogurt, through metabolites like SCFAs produced by intestinal microbiota, further contribute to its protective effect against allergies (81). Though observational, these studies hint at the importance of the maternal intake of fermented foods in preventing allergy outcomes in infants. Figure 2 summarizes the role of fermented foods in modulating the immune system during pregnancy and infancy.

## 6 Can fermented foods positively impact maternal and infant brain health?

Fermented foods are also psychobiotics due to their impact on brain outcomes (82, 83). The External Fermentation Hypothesis proposed by Bryant, Hansen and Hecht suggested that external fermentation practices could be the reason for the reduction of the human colon and expansion of brain volume due to decreased energy expenditure and increased accessibility of nutrients to the brain (84). Studies have suggested that fermented food can impact brain health throughout life, including early life brain development. Gut microbiota communicates with the central nervous system through three pathways, collectively comprising the “gut-brain axis” (85). In the first pathway, metabolites cross the blood–brain barrier and directly influence the central nervous system. In the second, they follow the same path but act by modulating the brain-resident immune cells. The third pathway is through the release of hormones from enteroendocrine cells (EECs) such as I cells, K cells and N cells during their interaction with microbes (86). During the early stages after birth, human milk feeding emerges as the most prominent and effective nutritional approach to support optimal brain development in infants and hence factors that determine the human milk composition play vital role (18). It is imperative to suggest that the gut microbiome modulating effect of fermented foods and its cascade effect on human milk components and infant gut microbiota assembly may be a precursor for early brain health in infants. Though this hypothesis sounds reasonable, most of the studies concerning the benefits of fermented foods on brain health were observed in adults and aging rodent models of neurological diseases, not in models of pregnancy.

Studies in adults and the aging population have shown that fermented foods can promote cognition, memory, learning, neurotransmitter production and overall brain health (87). In a randomized control trial, healthy women aged between 18 and 55 years were given fermented milk products in combination with four probiotics: *B. animalis, S. thermophiles, L. bulgaricus* and *L. lactis* daily for 4 weeks. In this study, the midbrain regions of women (that control emotion) were impacted, including reduced reactivity during emotional attention tasks (88). In clinical studies, consumption of *L. helveticus* fermented milk was reported to improve sleep efficiency, reduce wake episodes, and improve cognitive performance during cognitive fatigue tests in elderly subjects (89, 90). Similarly, fermented food also improved attention and memory in middle-aged Japanese subjects, potentially via a peptide, lactononadecapeptide (91). Other varieties of fermented foods, such as seaweed, yogurt, tofu, fermented soybeans and miso soup, were also found to be negatively associated with depression during pregnancy, which could mitigate the impact of maternal stress on the offspring (32).

**Figure 3:**
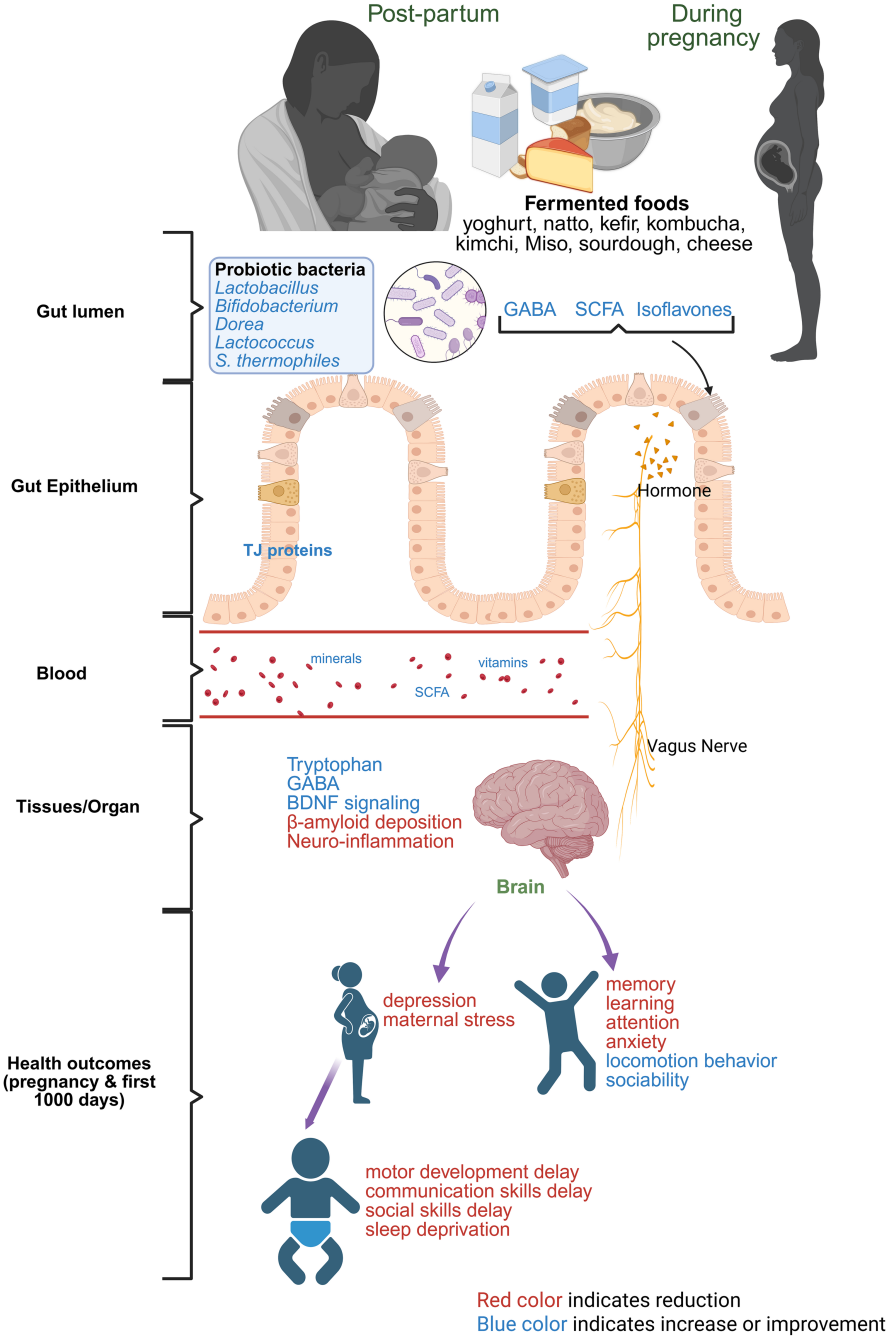
Role of fermented foods in aiding mother’s and infant’s brain health. Red color text indicates reduction and blue color text indicates improvement or amelioration due to maternal consumption of fermented foods.

Though limited, published literature has suggested the beneficial effect of maternal intake of fermented foods on offspring neurodevelopment (92, 93). For example, in a Japanese cohort, maternal consumption of fermented soybeans and miso soup from the beginning of pregnancy to the third trimester had a lower risk of fine motor development delay and communication skills delay in the 1-year-old offspring (93). In the same study, yogurt consumption by the mothers was associated with reduced personal and social skills delay in the offspring (93). In another Japanese study, consuming cheese during pregnancy was linked to a lower risk of developmental delays in children (92). Consuming miso soup during the second and third trimester of pregnancy was shown to reduce the risk of lower sleep hours in 1-year-old infants (94). A study on mother–child pairs indicated consuming fermented foods like cheese during pregnancy could reduce the risk of sleep deprivation in infants at 3 years of age (95). In a prenatal valproic acid-induced autism spectrum disorder mouse model, supplementation with *Lactiplantibacillus plantarum* fermented milk ameliorated some autism-like symptoms (improved locomotor behavior, sociability, anxiety) in male mice but not in female mice (96). Figure 3 summarizes the role of fermented foods in maternal and infant brain health. However, mechanistic studies on how maternal consumption of fermented foods program fetal brain and impact offspring’s brain development and cognitive measures are lacking. Similarly, studies on maternal and offspring consumption of fermented food during the postnatal period and the resulting behavioral and brain outcomes are limited. In summary, these reports underscore the benefits of fermented foods in alleviating brain-related disorders in adults and the aging population, but it is unknown whether fermented foods would have similar effects in pregnancy and these effects would be transgenerational. Exploring this would require placental and transgenerational animal models, especially to understand the role of maternal fermented food intake on the first 1,000 days on infant’s brain health and outcomes. Table 2 summarizes studies on consumption of fermented foods and the health outcomes during pregnancy and postnatal period.

**Table 2:** The role of fermented foods during pregnancy and infant’s first 1,000 days in relation to modulating gut microbiome, immune function and brain health.

Study details	Fermented food consumed	Study outcome and proposed mechanism	References
In humans			
Prospective cohort study conducted among pregnant women at risk for pre-term birth (*n* = 77,667)	Miso, natto, yogurt, fermented soybeans	Reduced risk of preterm birth due to lower composition of Enterobacteria associated with inflammation and immune dysregulation	(25)
Prospective cohort study conducted among pregnant women during mid-pregnancy (*n* = 965)	Yogurt	Reduced risk of preterm delivery due to plausible anti-inflammatory properties of probiotics	(26)
Cohort study among pregnant women between 5th and 39th week during pregnancy (*n* = 1,745)	Seaweed, yogurt, tofu, fermented soybeans and miso soup	Lowered depressive symptoms during pregnancy due to effects of soy isoflavones	(32)
Prospective cohort study conducted among women who gave birth (*n* = 9,030)	Miso, Natto, Yogurt, Fermented soybeans, Pickled vegetables	No strong association was observed between consumption of fermented foods and prevalence of psychological distress during pregnancy	(33)
Randomized controlled clinical trial (RCT) conducted among women during their third trimester (*n* = 70)	Yogurt	Prevention of pregnancy induced insulin resistance by stabilization of serum insulin levels	(41)
Observational case–control study among pregnant women (*n* = 123)	Yogurt	Reduced risk of gestational diabetes (GDM) via lowered blood glucose	(43)
Randomized crossover clinical trial among women with GDM (*n* = 62)	Whole grain sourdough bread	Lower postprandial blood glucose level with consumption of sourdough bread useful for dietary management of GDM	(42)
Randomized controlled clinical trial (RCT) among pregnant women (*n* = 84)	Yogurt containing *L. acidophillus* and *B. lactis*	Significant reduction in fasting plasma glucose levels	(44)
Prospective cohort study conducted among pregnant women (*n* = 56)	Yogurt	Higher relative abundance of fecal *Bifidobacterium* and a decrease in *Enterobacteriaceae* in infants; lowered blood glucose and HbA1c post-supplementation	(64)
Randomized, double-blind, placebo-controlled trial conducted among lactating mothers and their infants (*n* = 142)	Fermented milk	Increased CD4+ T-cell counts at week 6 in the treatment group compared to placebo; higher IL-10 production at weeks 3 and 6 in the treatment group; reduced TNF-α levels at week 6; higher sIgA concentrations in breast milk	(68)
Retrospective case-control study among infants aged 0–12 months and their mothers (*n* = 207)	Yogurt, cheese and tarhana	Lower consumption of yogurt, cheese, and tarhana were associated with increased FPIAP risk	(76)
Prospective cohort study in pregnant women and their infants aged 2 months to 2 years (*n* = 84)	Yogurt, cheese, kefir and pickles (olive)	Frequent consumption (≥3 times/week) of yogurt was associated with lower atopic dermatitis risk	(77)
Prospective cohort study conducted among mother and children aged till 36 months (*n* = 40,614)	Probiotic milk	Frequent maternal consumption reduced the risk for atopic dermatitis among infants	(78)
Prospective cohort study conducted among mothers and infants aged 6 months (*n* = 2,114)	Yogurt	Higher maternal yogurt consumption during pregnancy was associated with a reduced risk of infantile eczema (dose-dependent) through SCFA or anti-inflammatory properties of probiotics	(79)
Prospective cohort study conducted among pregnant women (*n* = 103,060)	Miso, Natto, Yogurt, Cheese	Cheese intake during pregnancy reduced developmental delay risk in offspring; miso and yogurt aided communication skills.	(93)
Prospective cohort study among mother–child pairs (*n* = 73,228)	Miso, Natto, Yogurt, Cheese	Cheese had the strongest protective effect against risk of developmental delay	(92)
Prospective cohort study among mother–child pairs (*n* = 72,626)	Miso, Natto, Yogurt, Cheese	Maternal miso consumption during pregnancy was independently linked to longer infant sleep duration at 1 year	(94)
Prospective cohort study among mother–child pairs (*n* = 68,255)	Miso, Yogurt, Cheese	Miso consumption during pregnancy was linked to longer child sleep duration at 3 years	(95)
In experimental animals			
Experimental animal trial in pregnant hypertensive rats (*n* = 16)	Buffalo yogurt	Mitigation of pregnancy-induced hypertension via ACE Inhibitory Peptides	(49)
Experimental animal trial in lactating Wistar rat dams (*n* = 24)	Kefir	Pups from kefir-fed dams exhibited a higher abundance of *Lactobacillus* and *Bifidobacterium* via vertical transmission from breast milk	(65)
Experimental animal study conducted in BALB/c mice (*n* = 60)	Fermented milk	Higher lactobacilli count in the intestines of pups whose mothers received fermented milk compared to controls; increase in lactobacilli population in intestine post-weaning	(67)
Experimental animal study conducted in pigs (*n* = 20) and C57BL/6 mice (*n* = 24)	Fermented corn & soybean	Reduced inflammatory response due to correlation with showed higher SCFA levels in colon	(69)
Experimental animal study conducted in germ-free C57BL/6 mice (*n* = 12)	Yogurt	Potential infection reduction through higher ILC3 populations in offspring intestines	(70)
Experimental animal study in Swiss albino mice (*n* = 12)	Fermented milk	Higher splenic IL-10 and IFN-γ; higher CD4 + T cells and CD19 + B cells post-weaning; elevated serum IgG in the treatment group	(71)
Experimental animal trial in offspring mice (*n* = 48) with induced autism	*L. plantarum* fermented milk	Improvement in autistic-like behaviors via the gut-microbiota-brain axis and SFCA production	(96)

## 7 Concluding remarks and future directions

The contemporary global health paradigm emphasizes a holistic approach to child development, shifting from mere survival to overall well-being, which is heavily influenced by maternal nutrition. The functional components of fermented foods, especially microbiome, can alter gut microbiota composition during pregnancy and in infants, thereby modulating their immune system and aiding brain outcomes. However, it is also important to note that though short-term dietary intervention can alter microbiota composition, changes are transient and do not last longer than a few days (97). This warrants an important question, “*Which period during pregnancy should supplementation of fermented foods begin to optimize positive health outcomes?”* Since most of the reported studies on the impact of fermented food are short-term, more long-term and longitudinal studies are needed to determine how long fermented food dietary intervention is required to alter the long-lasting microbial composition. Studies should also account for other potential confounding variables that may impact both microbiome composition and pregnancy outcomes. Our review highlights the benefits of maternal fermented foods intake on the offspring’s microbiota, immune system, and brain health. However, studies on fetal health, another critical period of development, are lacking which limits knowledge of causality. The review also highlights key pregnancy-related complications and the possible benefits of maternal consumption of fermented foods. However, the mechanistic pathways through which these effects are exerted need to be further examined, particularly considering risk factors like maternal age and pre-existing medical conditions. Further, the transgenerational effects of maternal intake of fermented foods on offspring remain largely unexplored. While methodological challenges in human cohort studies pose significant complexities, using animal models could bridge this knowledge gap. Finally, it should also be noted that considering the safety, demographic differences, cultural differences, dietary patterns and food systems, cost-effectiveness and broader economic feasibility of such interventions, we must be mindful of the “one size fits all” approach of touting fermented foods as a unique and gold standard intervention without having clear scientific consensus.
